# A novel *ETFB* mutation in a patient with glutaric aciduria type II

**DOI:** 10.1038/hgv.2015.16

**Published:** 2015-06-18

**Authors:** Yosuke Sudo, Ayako Sasaki, Takashi Wakabayashi, Chikahiko Numakura, Kiyoshi Hayasaka

**Affiliations:** 1 Department of Pediatrics, Yamagata University School of Medicine, Yamagata, Japan

## Abstract

Glutaric aciduria type II (GAII) is a rare inborn error of metabolism clinically classified into a neonatal-onset form with congenital anomalies, a neonatal-onset form without congenital anomalies and a mild and/or late-onset form (MIM #231680). Here, we report on a GAII patient carrying a homozygous novel c.143_145delAGG (p.Glu48del) mutation in the *ETFB* gene, who presented with a neonatal-onset form with congenital anomalies and rapidly developed cardiomegaly after birth.

Glutaric aciduria type II (GAII), also known as multiple acyl-CoA dehydrogenase deficiency, is an inborn error of metabolism clinically characterized by hypoketotic hypoglycemia and metabolic acidosis, pathologically by fatty infiltration of the liver, heart and kidneys, and biochemically by the accumulation of the metabolites of compounds catalyzed by enzymes that require electron transport flavoprotein (ETF) as an electron acceptor. GAII patients are clinically classified into three types: a neonatal-onset form with congenital anomalies, a neonatal-onset form without congenital anomalies and a mild and/or late-onset form (MIM #231680).^1,2^ The neonatal-onset form with congenital anomalies is lethal and is characterized by metabolic acidosis, hypoglycemia, hypotonia, ‘sweaty feet’ odor, facial dysmorphisms, polycystic kidneys, hepatomegaly and pulmonary hypoplasia.^2^ Some cases were noticed to have cardiomegaly after birth.^3^

ETF is a heterodimer of the ETF alpha subunit (ETFA) and ETF beta subunit (ETFB) that is located in the mitochondrial matrix and serves as the electron acceptor for at least nine flavoprotein dehydrogenases, including acyl-CoA dehydrogenases. It also enables both fatty acid beta-oxidation and amino acid catabolism within the main respiratory chain,^4^ and is reoxidized by ETF-ubiquinone oxidoreductase in the inner mitochondrial membrane. Most GAII patients have a defect in either ETF or electron transport flavoprotein dehydrogenase (ETFDH).

*ETFB* is located on chromosome 19q13.3 and consists of six exons. GAII patients carrying *ETFB* mutations can also be classified into the three different clinical forms mentioned earlier.^5,6^ Here, we present a GAII patient carrying a novel homozygous *ETFB* mutation, c.143_145delAGG (p.Glu48del), complicated with congenital anomalies and who rapidly developed cardiomegaly after birth.

The female GAII patient, a second child of healthy nonconsanguineous parents, was born at 31 weeks’ gestation by emergency cesarean section because of fetal distress. She had a healthy older brother and no family history of metabolic disease or sudden death in the neonatal period. At 21 weeks’ gestation, ultrasonography revealed that she had enlarged polycystic kidneys. Oligohydramnios gradually developed in the third trimester of pregnancy. Her birth weight was 1,962 g, and her Apgar score was 1 at 1 min and 3 at 5 min. Physical examination revealed low-set ears, micrognathia, redundant skin below the neck, a distended abdomen, a flexed position of both wrists, hypogenitalism and hypotonia.

Ultrasonography of the kidney and brain revealed bilateral enlarged hyperechoic kidneys and large cavum septi pellucidi, respectively. She received high-frequency oscillatory ventilation because of severe pulmonary hypoplasia soon after birth and peritoneal dialysis for profound oliguria 20 h after birth. She also received an intravenous glucose infusion (infusion rate: 10–12 mg/kg/min) after birth; however, severe hypoglycemia (blood glucose levels 16–34 mg/dl at 12–24 h old) was observed. She had persistent moderate metabolic acidosis despite treatment with sodium bicarbonate. At 2 days of age, she was noted to have a ‘sweaty feet’ odor and hyperammonemia (238 μmol/l). Urinary organic acid analysis revealed an increase in the excretion of adipic, suberic, sebacic, ethylmalonic, glutaric and fumaric acids and isovalerylglycine, suggesting a diagnosis of GAII. She was treated with intravenous riboflavin (100 mg/kg/d) and carnitine (100 mg/kg/d) administration. An echocardiogram revealed a slight thickening of the ventricular wall 3 days after birth, which rapidly increased as seen in hypertrophic cardiomyopathy. She developed cardiac dysfunction and died 6 days after birth. An autopsy revealed a thickened ventricle and numerous lipid droplets in the cardiomyocytes by Sudan III staining (Figures 1 and 2).

Molecular analysis of genomic DNA extracted from the peripheral blood cells of the patient and her parents was performed after informed consent was obtained. We PCR-amplified all coding regions of *ETFA* and *ETFB* using primers designed from genomic data. The patient was revealed to be a homozygote of the novel c.143_145delAGG (p.Glu48del) mutation in *ETFB*. Her parents were heterozygous for the mutation.

In GAII, lipid accumulation is observed in the tissues including the liver, heart and renal tubular epithelium, which use fatty acids as a primary source of energy. Colevas *et al.*^7^ speculated that the malformations might be the consequence of an accumulation of toxic metabolites that is not corrected by placental transfer. Enlarged polycystic kidneys and hepatomegaly are observed in many GAII patients and are often detected in prenatal periods.^8^ Cardiomegaly is also a complication of many patients, but is not frequently detected during prenatal periods despite common myocardial steatosis.^8,9^ Most GAII patients, including our case, rapidly develop cardiomegaly after birth. The major energy sources of the fetal heart are lactate and glucose, although this changes to fatty acids soon after birth.^10^ Such a change in the myocardial energy metabolism could explain the rapid development of cardiomegaly after birth and the absence of rapid cardiac change associated with neonatal-onset GAII.

Clinical phenotypes of GAII patients carrying *ETFB* mutations range from mild to lethal. Olsen *et al.*^6^ studied the genotype–phenotype relationship in GAII by expression experiments and found that null mutations caused the neonatal-onset form with congenital anomalies, whereas mutations resulting in some residual ETF/ETFDH enzyme activity were associated with a milder phenotype. Yotsumoto *et al.*^11^ studied 15 Japanese patients with GAII and detected three compound *ETFB* mutations in four patients: c.[78delG];[490C>T] (p.[Gly28fs];[Arg164Trp]) mutations in a patient with neonatal-onset GAII complicated with congenital anomalies; c.[55A>T];[81delC] (p.[Lys19*];[Gly28fs]) mutations in a patient with neonatal-onset GAII without congenital anomalies; and c.[491G>A];[597+1G>C] (p.[Arg164Gln];[Gly147_Met199del]) mutations in two patients with late-onset GAII. We speculate that the novel *ETFB* c.143_145delAGG (p.Glu48del) mutation would drastically change the EFTB structure leading to a marked decrease in ETF/ETFDH enzyme activity, as seen for null mutations.

In conclusion, we report the case of a severe form of GAII caused by a novel *ETFB* mutation. Information about genotype–phenotype relationships is important in the study of genetic disorders such as GAII.

## Figures and Tables

**Figure 1:**
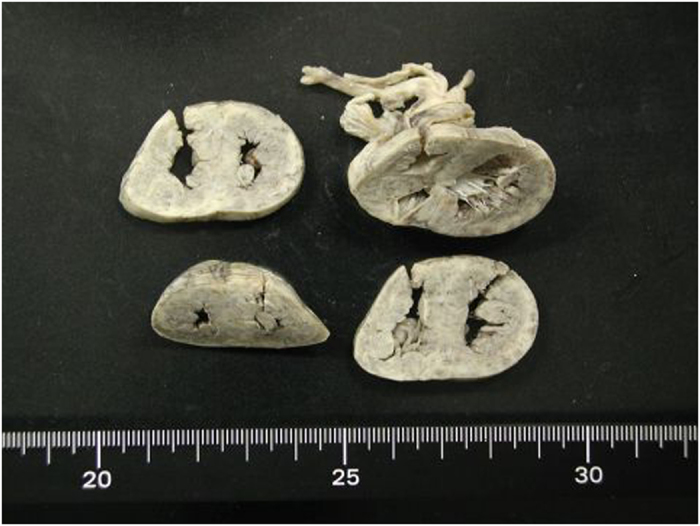
Heart of the 6-day-old GAII patient with a causative novel *ETFB* mutation showing a thickened ventricle wall and narrow ventricular lumen.

**Figure 2:**
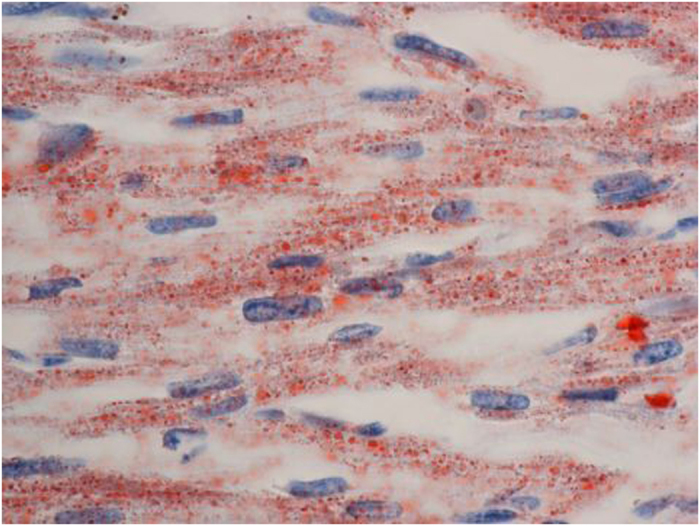
Fat droplet accumulation in cardiomyocytes by Sudan III staining. Original magnification ×400.
